# The challenge for general practitioners to keep in touch with vulnerable patients during the COVID-19 lockdown: an observational study in France

**DOI:** 10.1186/s12875-022-01694-y

**Published:** 2022-04-18

**Authors:** Tiphanie Bouchez, Sylvain Gautier, Julien Le Breton, Yann Bourgueil, Aline Ramond-Roquin

**Affiliations:** 1 University Côte d’Azur, Department of Education and Research in General Practice, RETINES, HEALTHY, F-06107 Nice, France; 2 Sorbonne University, University Paris Nord, Inserm, UMR_S 1142, LIMICS, Paris, France; 3 Paris-Saclay University, University of Versailles Saint-Quentin, Inserm U1018, CESP, Primary Care and Prevention, Villejuif, France; 4 Mission RESPIRE, EHESP-CNAMTS-IRDES - EA MOS 7348 EHESP, La Plaine Saint-Denis, France; 5 Department of epidemiology and public health, Hospital Raymond Poincaré, Assistance publique - Hôpitaux de Paris, Garches, France; 6 Univ Paris Est Creteil, INSERM, IMRB, CEpiA Team, F-94010 Créteil, France; 7 Univ Paris Est Creteil, Département de médecine générale, F-94010 Créteil, France; 8 Société Française de Médecine Générale (SFMG), Issy-les-Moulineaux, France; 9 Institut Jean-François REY (IJFR), Paris, France; 10 Institut de Recherches et de Documentation en Santé (IRDES), Paris, France; 11 Univ Angers, Département de médecine générale, F-49000 Angers, France; 12 Univ Angers, Univ Rennes, EHESP, Inserm, IRSET-ESTER, F-49000 Angers, France; 13 Collège National des Généralistes Enseignants (CNGE), Paris, France; 14 Département de médecine de famille et de médecine d’urgence, Université de Sherbrooke, Québec, Canada

**Keywords:** Family practice, Continuity of care, Vulnerability, Multidisciplinary care, Population health, Primary care, COVID-19

## Abstract

**Background:**

In France, the first COVID-19-related lockdown (17th March to 10th May 2020) resulted in a major decrease in healthcare service utilization. This raised concerns about the continuity of care for vulnerable patients.

**Objectives:**

To identify individual and organizational factors associated with the initiatives taken by French GPs to contact vulnerable patients during the lockdown.

**Methods:**

A national observational survey using an online questionnaire was conducted to document French GPs’ adaptations to the COVID-19 situation, their individual and organizational characteristics, including practice type (individual, group, multidisciplinary) and size. Data were collected from 7th to 20th May 2020 using mailing lists of GPs from the study partners and GPs who participated in a previous survey. This paper analysed answers to the question exploring whether and how GPs took initiatives to contact vulnerable patients. Responses were categorized in: no initiative; selection of patients to contact with a criteria-based strategy; initiative of contact without criteria-based strategy. Multivariate multinomial logistic regression identified factors associated with each category. Key components of the reported initiatives were described by inductive analysis of verbatim material.

**Results:**

Among the 3012 participant GPs (~ 5.6% of French GPs), 1419 (47.1%) reported initiatives to contact some patients without criteria-based strategy, and 808 (26.8%) with a strategy using various clinical/psychological/social criteria. Women GPs more often declared initiatives of contacts with a criteria-based strategy (OR = 1.41, 95% CI [1.14-1.75]) as well as GPs with more than two patients who died due to COVID-19 in comparison with those having none (OR = 1.84, 95% CI [1.43-2.36]). Teaching GPs more often used criteria-based strategies than the other GPs (OR = 1.94, 95% CI [1.51-2.48]). Compared with those working in small monodisciplinary practice, GPs working alone were less likely to implement criteria-based initiatives of contacts (OR = 0.70, 95% CI [0.51-0.97]), while GPs working in multidisciplinary practice were more likely (OR = 1.94, 95% CI [1.26-2.98] in practices > 20 professionals).

**Conclusion:**

French GPs took various initiatives to keep in touch with vulnerable patients, more frequently when working in group practices. These findings confirm the importance of primary care organization to ensure continuity of care for vulnerable people.

**Supplementary Information:**

The online version contains supplementary material available at 10.1186/s12875-022-01694-y.

## Introduction

In May 2020, France ranked sixth in the world for the number of confirmed cases of COVID-19 [1]. In a context of shortage of personal protection equipment and limited testing [2], the French government put in place a strict population lockdown from 17 March 2020 to 11 May 2020. Although health issues were explicitly listed as one of the few compelling reasons to leave the house, healthcare service utilization dramatically decreased during the lockdown. In May 2020, the number of general practice consultations was still 20% below the usual number, despite the deployment of teleconsultation [3]. Concerns emerged about the consequences of this reduced care utilization [4–7]. Therefore, in April 2020, the French government invited the population to seek care as much as they needed, and asked physicians, especially general practitioners (GPs), to contact patients with chronic diseases [8].

In France, primary healthcare actors have been implicated in the COVID-19 pandemic management during the mitigation strategy, but in a less coordinated manner than hospitals. This could be explained by the fact that French primary care is mainly organized as private, independent services, although financed by the public health insurance. Like most primary care providers in France, GPs work mainly in private practices and are paid on a fee-for-service basis. However, many reforms have been implemented in primary care in the last 30 years. In 2004, a preferred doctor scheme was introduced with success because almost 80% of the population is now registered with a GP [9]. Group practice is now dominant among GPs and 81% of < 50-year-old GPs declare working in group practices (mono or multidisciplinary) [10]. Since the 2000s, teamwork has been developed, mainly in under-deserved areas to maintain primary care services [11]. Two main primary care multidisciplinary practice types co-exist in France. In independent multidisciplinary groups (*n =* 1617 in France), most professionals are independent, while in care centres (*n =* 428 in France), professionals are usually salaried. In both systems, professionals agree on common health-related objectives and collective actions for the population they care for and may choose to sign a contract with the regional health authority to obtain financial resources for collective actions. The engagement of primary care providers toward social responsibility [12] officially started with the introduction of a meso-level organization named “integrated territorial professional community” in the 2016 law [13]. Therefore, the COVID-19 pandemic occurs in a primary care system that is moving from a curative and reactive practice by a single GP to a more integrated, preventive, and proactive practice. Moreover, French GPs are not used to reach out to patients because normally patients come to their GP for a specific problem or for a follow-up. Consequently, no system was in place for systematically contacting patients.

In this context, an emerging multidisciplinary primary care research network (ACCORD) carried out several surveys to explore various primary care providers’ adaptations to the COVID-19 pandemic situation and to identify individual, organizational, and territorial factors associated to these adaptations, with a special interest for organizational factors [14]. Two successive surveys (at the beginning, then at the end of the first lockdown) aimed at documenting GPs’ adaptations related to different domains, one of these being continuity of care for vulnerable patients. The objectives of the present paper were (i) to describe whether and (ii) how French GPs took initiatives for identifying and contacting vulnerable patients during the first lockdown, and (iii) to identify individual and organizational factors associated with these initiatives.

## Methods

### Study design

This paper builds on selected data collected during the second national observational survey conducted among GPs in France by ACCORD network. This survey was aimed at documenting adaptations of GPs at the end of the first lockdown in France (May 2020) and identifying individual, organizational and territorial factors associated to these adaptations, with a special interest for organizational factors. It was based on a questionnaire that could be filled in on-line using the free *LimeSurvey* tool. The questionnaire was created by the survey team members, inspired by clinical experience-based hypotheses, available literature, and answers to the previous survey (March 2020) [14], then revised by a panel of 7 primary care experts and finally piloted by the survey team members before the survey being launched. A message with the link to the survey was sent using national mailing lists of GPs (N ~ 25,000) from the study partners (see Additional file 1) and the mailing list of GPs (*N* = 4436) who participated in the previous survey organized in March 2020 by the same partners [14]. The link to the survey was also disseminated using social networks. Participants were invited to send the survey link to their colleagues. After reading information concerning aims, methods, sources of funding, institutional affiliations of the researchers and the anticipated benefits, subjects gave their informed consent to participate by clicking and answering the questionnaire. Data were collected between 7th and 20th May 2020.

### Data

The questionnaire for this survey was composed of 63 questions. A first part explored adaptations to the pandemic situation in relation with 7 domains of interest (activity, prescriptions, occupational health, patients with COVID-19, nursing home residents, vulnerable patients, territorial partnerships). The second part collected GP’s individual characteristics (gender, age, teaching activity, other complementary clinical activities, usual density of activity, recent quantitative activity changes, number of patients hospitalized for/who died due to COVID-19, being at risk of severe COVID-19, fear of SARS-COV2), organizational characteristics (type of practice: alone/monodisciplinary practice/independent multidisciplinary group/care centre; size of practice) and territorial characteristics (location of the practice, relations with the hospital, local partners and networks).

This paper specifically builds on the answers to the following question (thereafter called “question of interest”): “Did you take the initiative to contact by phone some of your patients (e.g. vulnerable, with chronic diseases)?” with three possible answers: “No, I did not call any patient”, “Yes, the ones I thought about”, and “Yes, I made a list using some criteria (e.g. patients with 100% coverage by the French national insurance due to a chronic disease, body mass index)”. A fourth answer choice was also proposed: “Other, please specify”. These latter answers were manually recoded into one of three previously described answer categories when the verbatim was explicit enough (See Table 1). In the other cases, observations were excluded and considered as missing data for the question of interest.

**Table 1:** Classification of the answers to the question of interest

Question of interest: “Did you take the initiative to contact by phone some of your patients (e.g. vulnerable, with chronic diseases)?”			
Possible answers	Number of respondents (*n =* 3030)	Three categories	Dichotomous variable
“No, I did not call any patient”	761	“No initiative”	No
“Yes, the ones I thought about”	1416	“Yes, without criteria-based strategy”	Yes
“Yes, I made a list using some criteria (e.g. patients with 100% coverage by the French national insurance due to a chronic disease, body mass index)”	730	“Yes, with criteria-based strategy”	
“Other, please specify”	123	Classified into one of the previous three categories*	*Not concerned*

*no initiative: *n =* 24; yes, without strategy: *n =* 3; yes, with strategy: *n =* 78. missing data: *n =* 18

### Inclusion and exclusion criteria

Incomplete questionnaires and duplicates were excluded. The questionnaire was considered complete if including at least the GPs adaptations as well as organizational and territorial factors, age and sex. The duplicates were identified searching a same token (individual number of access to the questionnaire for the responders to the previous study who agreed to be surveyed again), registration number in the national directory of health professionals or email address. The sample was then limited to GPs practicing in Metropolitan France. Questionnaires from GPs who declared no clinical activity in the last 7 days were finally excluded because these GPs were considered not to have a good insight into practice adaptations in a quickly evolving situation, as well as those with missing data for the question of interest.

### Data recoding

In the first analyses the variable of interest was considered as a dichotomous variable Yes/No; thus, the two answer categories “Yes, without criteria-based strategy” and “Yes, with criteria-based strategy” were considered together as “Yes”.

For the final model, independent multidisciplinary group and care centre were merged into one modality: “multidisciplinary practice”. Moreover, the practice type and practice size were expected to be highly collinear. As both were considered very relevant for the question of interest, a composite variable was constructed rather than favouring one over the other. Therefore, a new variable “type and size of practice” was created with five modalities: i) alone, ii) monodisciplinary practice with 5 ≤ professionals, iii) monodisciplinary practice with > 5 professionals, iv) multidisciplinary practice with ≤20 professionals, and v) multidisciplinary practice with > 20 professionals.

### Quantitative data analyses

The GPs’ characteristics were first described. Then, bivariate analyses were carried out using the question of interest considered as a dichotomous variable, followed by bivariate analyses using the question of interest considered in three answer categories, using chi-2 tests for categorical data. Then, an unordered multivariate multinomial logistic regression model was used to assess the relative contribution of the different factors. To assess the potential role of all variables of interest, no selection of variables was undertaken. The reference categories were commonly those with the larger population, except for age and number of patients who died of COVID-19 for which we tested specific hypothesis. For age group, in the demographic context of the current switch of the older generation by a younger workforce, we explored the effect of being among GPs in their first 10 years of practicing and of being in their last 10 years of practicing, versus reference category of 40-55 years. The likelihood ratio chi-square and the Score and Wald tests were used to assess how well the multivariate model fitted the data. SAS software (version 9.4) was used to undertake these analyses and the statistical significance threshold was set at 5%.

### Complementary qualitative data analyses

A qualitative analysis of the verbatim material for the answer “Other, please specify” was performed to explore key components of the reported contact initiatives. A general inductive approach was used to extract units of meaning and articulate emerging concepts. It consisted of open labelling followed by categorization [15], using NVivo10®. The same verbatim section could be labelled with more than one unit of meaning. The analysis was performed by one of the authors (TB) and the final categorization was generated during two consensus meetings with the whole research group.

## Results

### Sample characteristics

**Fig. 1:**
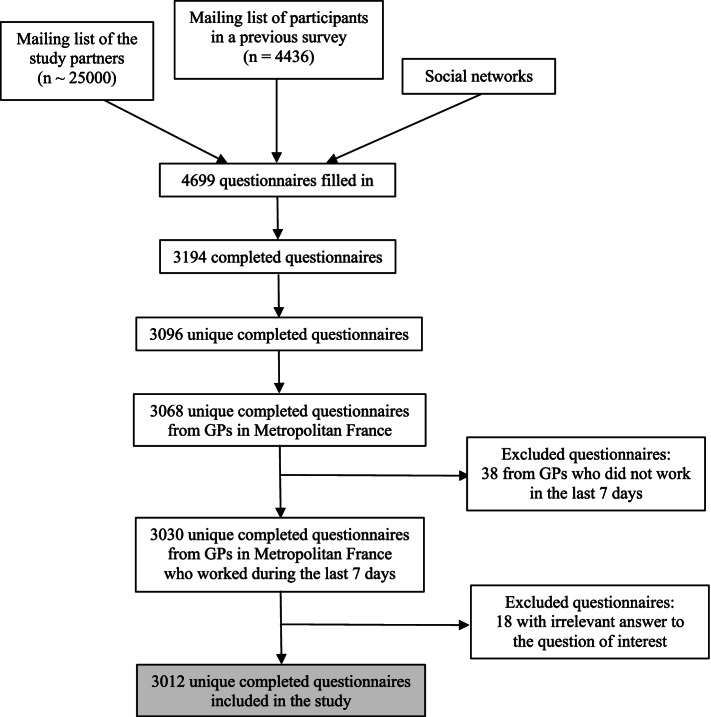
Flowchart of the study on patient-contacting initiatives among GPs in France in May 2020 during the COVID-19 lockdown: 3012 questionnaires were retained for the analyses

Among the 4699 questionnaires that were filled in, 3096 were retained after exclusion of duplicates and incomplete questionnaires. Among the 3068 (99.1%) questionnaires by GPs practicing in Metropolitan France, 38 were excluded because these GPs declared no clinical activity in the last 7 days and 18 because of missing data for the question of interest, resulting in a final sample of 3012 questionnaires (Fig. 1). Among this study sample, 1659 (55.1%) were women, and 1127 (37.5%) participants were younger than 40 years of age. Moreover, 469 (15.6%) participants declared that they worked alone, 1300 (43.4%) in a monodisciplinary practice, and 1228 (41.0%) in a multidisciplinary practice (*n =* 1099, 89.5%, in an independent multidisciplinary group and 129, 10.5%, in a care centre) (Table 2). The sample corresponds to 5.6% (3012/53,339) of all currently active French GPs [16]. Responders were younger (< 40 years, 37.6% vs 17%), more frequently women (55.1% vs 44%) [16] and worked less frequently alone (15.6% vs 39%) [10] than the whole French GPs’ population (Table 2). All French regions were represented (Additional file 2).

**Table 2:** Main characteristics of the survey participants (*n* = 3012) compared with general practitioners’ population in metropolitan France in 2020

n (%)	Study sample *n =* 3012	GPs’ population in metropolitan France^a^ *n =* 53,339
Gender		
Women	1659 (55.1)	23,576 (44.2)
Men	1353 (44.9)	29,763 (55.8)
Age group		
< 40 years	1127 (37.5)	9068 (17.0)
[40-55[	864 (28.8)	15,255 (28.6)
> 55 years	1010 (33.7)	29,016 (54.4)
*Missing data*	11	
Type of practice		
Alone	469 (15.6)	20,802 (39.0) ^b^
Monodisciplinary practice	1300 (43.4)	
*Only GPs*	*1228 (41.0)*	
*GPs and other specialists*	*72 (2.4)*	
Multidisciplinary practice	1228 (41.0)	
*Independent multidisciplinary group*	*1099 (36.7)*	
*Care centre*	*129 (4.3)*	
*Missing data*	*15*	

GP: general practitioner

^a^Data from the French health insurance system (CNAMTS) (2019)

^b^Data from the French direction of research, studies, evaluation and statistics (DREES) (2019)

### Quantitative results

#### Question of interest considered as a dichotomous variable

Concerning the question of interest “Did you take the initiative to contact by telephone some of your patients (e.g., vulnerable, with chronic diseases)?”, 2227 (73.9%) participants gave a positive answer and 785 (26.1%) a negative answer. Bivariate analyses revealed that being a woman, teaching activity, having more patients hospitalized for/who died due to COVID-19, and working in group, especially in a care centre, were associated with a higher probability of contact initiatives, while a major decrease of activity during the last 7 days and working alone were associated with a lower probability (*p* <  0.05, Table 3).

**Table 3:** Individual and organizational factors potentially associated with the initiative of contacting vulnerable patients during the first COVID-19 lockdown in 2020 (considered as a dichotomous variable and in three categories): bivariate analysis (*n =* 3012 French general practitioners)

Bivariate analysis with initiatives considered as:		Dichotomous variable		Variable in 3 categories		
n (%)	**No initiative (reference)** *n =* 785	**Yes, with or without criteria**^a^ *n =* 2227	*p*^b^	**Yes, without criteria**^a^ *n =* 1419	**Yes, with criteria**^a^ *n =* 808	*p*^*c*^
Gender						**< 0.01**
Women	394 (23.7)	1265 (76.3)	**0.001**	799 (48.2)	466 (28.1)	
Men	391 (28.9)	962 (71.1)		620 (45.8)	342 (25.3)	
Age group						**0.01**
< 40 years	314 (27.9)	813 (72.1)	0.11	515 (45.7)	298 (26.4)	
[40-55[	205 (23.7)	659 (76.3)		396 (45.8)	263 (30.4)	
> 55 years	264 (26.1)	746 (73.9)		503 (49.8)	243 (24.1)	
*Missing data*	*2*	*9*		*5*	*4*	
Teaching activities						**< 0.01**
Yes	501 (24.4)	1556 (75.6)	**0.002**	926 (45.0)	630 (30.6)	
No	284 (29.7)	671 (70.3)		493 (51.6)	178 (18.6)	
Complementary activity in local hospital						0.06
Yes	48 (25.4)	141 (74.6)	0.83	77 (40.7)	64 (33.9)	
No	737 (26.1)	2086 (73.9)		1342 (47.5)	744 (26.4)	
Complementary activity as nursing home manager						**0.04**
Yes	46 (33.3)	92 (66.7)	0.05	66 (47.8)	26 (18.8)	
No	739 (25.7)	2135 (74.3)		1353 (47.1)	782 (27.2)	
Usual annual activity						0.27
< 3500 encounters per year	144 (25.4)	424 (74.6)	0.58	253 (44.5)	171 (30.1)	
Between 3500 and 6000	487 (25.8)	1403 (74.2)		902 (47.7)	501 (26.5)	
> 6000 encounters per year	154 (27.8)	400 (72.2)		264 (47.7)	136 (24.6)	
Usual daily activity						0.81
< 20 patients	105 (26.9)	285 (73.1)	0.77	177 (45.4)	108 (27.7)	
[20-30[	592 (25.7)	1710 (74.3)		1086 (47.2)	624 (27.1)	
> 30 patients	84 (27.3)	224 (72.7)		149 (48.4)	75 (24.4)	
*Missing data*	*4*	*8*		*7*	*1*	
Quantitative change of activity (last 7 days)						0.79
< 50%	128 (26.8)	150 (54.0)	**< 0.001**	22 (46.4)	128 (26.8)	
50 to 99%	406 (26.0)	1157 (74.0)		749 (47.9)	408 (26.1)	
Same number	208 (26.1)	587 (73.8)		359 (45.2)	228 (28.7)	
More patients	43 (24.4)	133 (75.6)		89 (50.6)	44 (25.0)	
Number of patients hospitalized for COVID-19						**< 0.01**
None	292 (29.7)	691 (70.3)	**0.003**	448 (45.6)	243 (24.7)	
1-5	391 (24.7)	1192 (75.3)		765 (48.3)	427 (27.0)	
> 5 patients	95 (22.1)	334 (77.9)		199 (46.4)	135 (31.5)	
*Missing data*	*7*	*10*		*7*	*3*	
Number of patients who died due to COVID-19						**< 0.01**
None	292 (29.7)	691 (70.3)	**< 0.01**	448 (45.6)	243 (24.7)	
1-2 patients	261 (27.7)	681 (72.3)		436 (46.3)	245 (26.0)	
> 2 patients	225 (21.0)	845 (79.0)		528 (49.4)	317 (29.6)	
*Missing data*	*7*	*10*		*7*	*3*	
Type of practice						**< 0.01**
Alone	146 (31.1)	323 (68.9)	**< 0.01**	231 (49.3)	92 (19.6)	
Monodisciplinary practice	338 (26.0)	962 (74.0)		654 (50.3)	308 (23.7)	
Independent multidisciplinary group	278 (25.3)	821 (74.7)		460 (41.9)	361 (32.9)	
Care centre	18 (14.0)	111 (86.0)		67 (51.9)	44 (34.1)	
*Missing data*	5	*10*		*7*	*3*	
Size of practice						**< 0.01**
Alone	146 (31.1)	323 (68.9)	**0.04**	231 (49.3)	92 (19.6)	
< 5 professionals	322 (25.6)	937 (74.4)		634 (50.4)	303 (24.1)	
[6-20[	271 (25.0)	813 (75.0)		478 (44.1)	335 (30.9)	
> 20 professionals	45 (22.6)	154 (77.4)		76 (38.2)	78 (39.2)	
*Missing data*	*1*	*0*		*0*	*0*	

^a^criteria is a shortcut for “criteria-based strategy”

^b^comparison between “no initiative” and “initiative with or without criteria-based strategy” (dichotomous variable)

^c^comparison between “no initiative”, “initiative with criteria-based strategy” and “initiative without criteria-based strategy” (3-category variable)

*p values <  0.05 are bolded*

#### Question of interest classified in three categories

Among the 3012 GPs, 1419 (47.1%) said that they phoned the patients they thought about (“no criteria-based strategy”), 808 (26.8%) phoned patients identified using some criteria (“criteria-based strategy”), and 785 (26.1%) declared no initiative of contact.

Bivariate analysis (Table 3) showed that the GPs who used a criteria-based strategy more often were in the 40 to 54 years age group, had teaching activities, and ≥ 5 patients hospitalized for COVID-19 (*p* <  0.01). Considering the practice type, there was a gradient of criteria-based strategy use. Specifically, 19.6, 23.7, 32.9, and 34.1% of GPs working alone, in monodisciplinary practices, in independent multidisciplinary groups, and in care centres reported using a criteria-based strategy (*p <* 0.01). Considering the practice size, 24.1, 30.9 and 39.2% of GPs working in a structure with 2 to 5, 6 to 20 and with ≥20 professionals, respectively, reported criteria-based strategies (*p <* 0.01).

Measures of multivariate model goodness of fit were satisfactory, with a likelihood ratio chi-square of 163.25 (*p* <  0.0001, as were the Score and Wald tests). Multivariate analysis (Table 4) confirmed that gender, teaching activities, number of patients who died due to COVID-19, and practice type and size were independently and significantly associated with contact initiatives. Conversely, age, complementary activity (in local hospital or as nursing home manager), usual activity density, and recent quantitative change of activity were not. Specifically, women GPs were more likely to take the initiative to contact patients, with and without criteria-based strategy (adjusted odds ratio, aOR = 1.41, 95% CI [1.14-1.75] and aOR = 1.37, 95% CI [1.13-1.66], respectively) (*p* = 0.001), while GPs with teaching activities were nearly 2 times more likely to use a criteria-based strategy (aOR = 1.94, 95% CI [1.51-2.48], *p <* 0.001). Having more than two patients who died due to COVID-19 increased by about 60 and 84% the probability of contacting patients without and with a criteria-based strategy (aOR = 1.60, 95% CI [1.28-1.99], and aOR = 1.84, 95% CI [1.43-2.36], respectively) (*p <* 0.001). Moreover, working in a multidisciplinary practice was significantly associated with the use of a criteria-based strategy to contact patients (aOR = 1.33, 95% CI [1.04-1.69] in multidisciplinary practice with 2 to 20 professionals; aOR = 1.94, 95% CI [1.26-2.98] in structures with ≥20 professionals). Conversely, GPs who worked alone were about 30% less likely to contact their patient during the lockdown, with and without criteria-based strategy (aOR = 0.72, 95% CI [0.55-0.93] and aOR = 0.70, 95% CI [0.51-0.97], respectively).

**Table 4:** Individual and organizational factors potentially associated with the initiative of contacting vulnerable patients during the first COVID-19 lockdown in 2020: multivariate multinomial logistic model (total *n =* 3012 French general practitioners)

n (%)	Call without criteria-based strategy *n =* 1419		Call with criteria-based strategy *n =* 808	
	aOR (95CI)	*p* value	aOR (95CI)	*p* value
Women	1.37 (1.13-1.66)	**< 0.001**	1.41 (1.14-1.75)	**0.001**
Age group				
< 40 years	0.80 (0.63-1.01)	0.063	0.79 (0.61-1.03)	0.083
[40-55[	Ref	–	Ref	–
> 55 years	1.07 (0.84-1.35)	0.60	0.83 (0.63-1.08)	0.16
Teaching activities	1.00 (0.81-1.22)	0.98	1.94 (1.51-2.48)	**< 0.001**
Complementary activity in local hospital	0.81 (0.54-1.23)	0.32	0.97 (0.62-1.50)	0.88
Complementary activity as nursing home manager	0.93 (0.75-1.15)	0.51	0.81 (0.64-1.03)	0.090
Usual annual activity				
< 3500 encounters per year	0.97 (0.76-1.23)	0.78	1.22 (0.93-1.60)	0.15
Between 3500 and 6000	Ref	–	Ref	–
> 6000 encounters per year	0.92 (0.73-1.17)	0.51	0.84 (0.63-1.11)	0.21
Quantitative change of activity (last 7 days)				
< 50%	0.94 (0.73-1.21)	0.63	1.11 (0.83-1.49)	0.48
50 to 99%	Ref	–	Ref	–
Same number	0.92 (0.74-1.14)	0.44	0.99 (0.78-1.26)	0.91
More patients	0.98 (0.67-1.45)	0.93	0.90 (0.57-1.42)	0.65
Number of patients who died due to COVID-19				
None	Ref	–	Ref	–
1-2 patients	1.10 (0.88-1.36)	0.41	1.19 (0.93-1.53)	0.17
> 2 patients	1.60 (1.28-1.99)	**< 0.001**	1.84 (1.43-2.36)	**< 0.001**
Type and size of practice				
Alone	0.72 (0.55-0.93)	**0.014**	0.70 (0.51-0.97)	**0.030**
Monodisciplinary practice with 2-5 professionals	Ref	–	Ref	–
Monodisciplinary practice with > 5 professionals	0.73 (0.49-1.07)	0.10	0.98 (0.63-1.51)	0.91
Multidisciplinary practice with 2-20 professionals	0.88 (0.71-1.08)	0.22	1.33 (1.04-1.69)	**0.022**
Multidisciplinary practice with > 20 professionals	0.99 (0.65-1.50)	0.94	1.94 (1.26-2.98)	**0.0026**

*p values* < 0.05 are bolded

### Complementary qualitative results

**Fig. 2:**
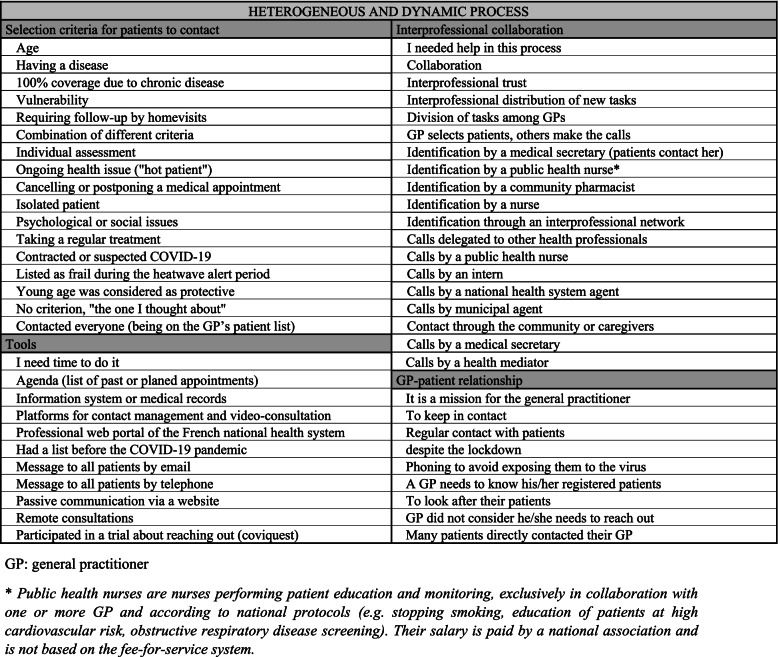
Results of the qualitative analysis on the initiatives taken by 123 general practitioners in France to get in touch with vulnerable patients during the first COVID-19 lockdown in 2020

Among the 3012 responding GPs, 123 (4.1%) filled in the “Other, specify” text field. The inductive analysis of the 115 unique answers resulted in 58 labels, classified in four categories (Fig. 2), and articulated in three concepts: “vulnerability” “organization” and “mission”. **The concept of vulnerability** covered a wide range of patients’ selection criteria indicated by GPs. It gave insights into a composite and empirical definition of vulnerability in the COVID-19 pandemic context: age, ongoing disease or follow-up, administrative criteria about chronic disease coverage, limited mobility, social criteria. Many GPs described a multicriteria approach, sometimes with individual assessments. Some said “the one I thought about”, highlighting the inconscient process of assessment based on multiple “gut” factors. This “human brain” tool seemed important. Indeed, some young GPs said that they felt helpless because “they did not know the patients”. Very few had a pre-existing list of vulnerable patients. Some simply contacted all their patients. The concept of organization covered the resources mobilized by GPs to contact their patients. Technical tools and collaboration were mentioned as resources first to identify vulnerable patients, and then to contact them. Most respondents described the use of their agenda to list the patients they met the past months or were supposed to meet during the lockdown. Fewer used medical records or national health system data to identify vulnerability criteria. Many GPs also described an informal interprofessional network (e.g., nurses, community pharmacists, medical secretaries) to identify patients. To contact patients, GPs used different strategies: collective contacts (email messages, texts, or website pages), but mainly individual contacts (telephone, video consultations). They often reported relying on collaboration with medical secretaries, medical students, nurses and especially public health nurses, associate GPs, or informal caregivers to perform the actual contact. Mission was the emerging concept about the GP-patient relation. Respondents described their initiative as a mission and more than a one-call procedure. They wrote about regular contacts, closer to a guardian role. Their motivation for this remote contact was to avoid any physical contact and the related risk of infection for their patients. They wrote about “keeping in contact”, an almost physical expression, while the questionnaire used the technical word “phoning”. However, some considered that their complete availability was sufficient to ensure that patients would contact them if needed (as usually done) and did not call them.

Finally, the analysis of the collected data indicated a significant variability and a dynamic process. Keeping in touch during the lockdown was an evolving task, in a rapidly changing environment.

## Discussion

The main results of this study were that (i) almost 74% of the retained French GPs who filled in the survey declared that they took initiatives to contact some patients; (ii) ~ 27% of them reported using various criteria and tools to identify vulnerable patients and/or relied on interprofessional collaboration; (iii) women GPs, GPs with patients who died due to COVID-19, and GPs not working alone took initiatives more often. Moreover, teaching GPs and GPs working in a multidisciplinary practice used criteria-based strategies more often.

In our study, many respondents considered essential to proactively reach out to vulnerable patients and assumed this unusual role. This might reflect an early awareness by healthcare professionals of the possible collateral damages of lockdowns in vulnerable people, such as renouncing care or care delay, as already highlighted in the literature [17]. Studies are still needed to determine whether getting in touch with vulnerable patients limited damages.

Most GPs had no specific strategy and called the patients “they thought about”. In addition to older adults and patients with chronic diseases, they especially called patients in situations of isolation, which is a risk factor of mental health deterioration, particularly during a lockdown [18]. However, they often excluded younger patients, although they also may be at risk of renouncing care [6] and mental problems. This raises questions about the criteria and the tools used to identify vulnerable patients. Our results suggest that GPs used various criteria related to biomedical, psychological, and social dimensions of health, in agreement with the multidimensional concept of vulnerability [19]. However, the GPs’ contact practices were heterogeneous and probably resulted from different conceptions of vulnerability.

Our study also showed that the GP’s knowledge as the only resource for targeting patients may be insufficient. Indeed, recently graduated GPs described the impossibility to identify vulnerable people among patients they do not know well yet. In France, medical records are usually comprehensive in terms of biomedical data. Conversely, information on psychological problems is often incomplete and social data are frequently missing. Besides age, medications, and comorbidities, social characteristics (i.e., living arrangement, mood, nutrition, mobility, autonomy, financial situation, and health literacy) could be objectified with tools validated in the context of primary care ( [20, 21]). The collection of such data in the context of general practice consultations seems to be well accepted by GPs and patients in France, although it requires time and communication skills [22]. However, even when such data are present in the records, the tools, and skills to perform routine data screening are lacking [23]. This exceptional context may be an opportunity for GPs and multidisciplinary primary care practices to develop and put in place the tools necessary for targeting and reaching specific patients within their patient database.

Among the study strengths, the sample size was large, with more than 3000 participating GPs (> 5% of the whole population of French GPs). Although some of the confidence intervals may appear somewhat large, this sample size reached the objective of identifying clinically and statistically significant associations. In addition, all metropolitan French regions were represented. We followed a data cleaning process, based on rigorous quality controls related to several key variables, to exclude duplicates and/or incomplete responses. The quantitative data analysis was robust, using validated modelling tools to produce multivariate results. The qualitative analysis results offered a deeper understanding of the issue under study. Although the description of the initiatives was limited to the survey material without complementary interviews or observations, the verbatim was explicit and resulted in rich and meaningful results. Finally, our findings represent an early and rare insight into practice and adaptations of French primary care providers during the COVID-19 pandemic, when research activities were under constraint, especially in primary care where information system is lacking.

This study has however some limitations. First, our sample is not representative of all French GPs because the study participants were younger, more frequently women and worked less frequently alone. These three variables are linked because in France, young GPs are more often women and work more often in group. Teaching GPs also were over-represented (68.3% in our sample versus ~ 20% of the whole French GPs’ population), because of the sampling strategy. Second, it was a declarative survey, and the answers might have been influenced by a social desirability bias. This may result in an overestimation of the real proportion of GPs who have taken initiatives to contact vulnerable patients. Our findings are close to the results issued from a survey on a representative panel of French GPs undertaken from the 9th to the 21st of April 2020 [24] which concluded that half of the GPs reached out to their vulnerable patients. The difference could be explained not only by the bias of our sample but also by the difference between the two survey periods, while GPs practices evolved quickly.

Beyond the estimation of the proportion of GPs involved in initiatives of contacts, the main contribution of our results is in the associations identified between these initiatives and some individual and organisational factors. Regarding the individual factors, we found that a bit more women GPs outreached to patients (73.3% versus 71.1% men) while no difference was observed according to GPs’ age. These results are similar to those of the survey previously mentioned [24]. In addition, GPs with a teaching activity were more likely to use a patient selection strategy. This could be explained by the fact that on 23 March 2020, the National College of Academic GPs (CNGE) already incited its members to pro-actively organize the continuity of care for their vulnerable patients. In addition, it is known that the practices and patients of training GPs slightly differ from those of non-training GPs, particularly better performances in diabetes follow-up, seasonal flu vaccination and breast cancer screening rates and lower rates of patients with low income [25]. Other associated factors were contextual: GPs with patients who died due to COVID-19 called their patients more often, possibly due to an increased awareness of their vulnerability. Regarding organisational factors, the independent association we identified between type and size of practice and GPs’ capacity to adapt practices and to implement new and complex tasks had not yet been described to our knowledge. Independent multidisciplinary groups or care centres are places of innovation in primary care, such as shared medical records systems, time of coordination and therapeutic education. The presence of different professionals, students, and in some cases public health nurses, facilitate the emergence of more structured strategies. Previous research [23] demonstrated the efficiency of such settings for the management of chronic patients [26]. If COVID-19 is considered a syndemic [27], multidisciplinary practice in primary care appears to be a valid model to respond to future needs and risks. Collaboration with other professionals (especially nurses, but also medical assistants, community pharmacists, etc.) through interprofessional networks was also cited by respondents as helpful to identify and to contact vulnerable patients. Interprofessional collaboration, within multidisciplinary practices but also at the local territorial level, might constitute a relevant strategy to support the implementation of such proactive practice. Future studies should explore the effectiveness of these emerging initiatives.

A recent review of evidence from past epidemics identified key lessons for primary care, including: improving collaboration, communication and integration between public health and primary care actors; strengthening the primary healthcare system; and defining the role of primary care during pandemics [28]. Our study confirms the importance of collaboration among healthcare professionals and of organized multidisciplinary practices to enhance the role that GP’s can take at the intersection between primary care and public health approach. Due to the specific sample characteristics, our results may reflect the practice and adaptations of a population of French GPs particularly involved in innovative practices and teamwork. Considering that practices of GPs involved in teaching activities have the potential of influencing practices of future generations of GPs, our results may also offer a projected vision of the primary care workforce on which the French primary care system could capitalize in the future [29]. Relying on the growing generation of young professionals who prefer collective practice [10], public health policy in France should accelerate the diffusion and role of multidisciplinary practices in the global care management of populations.

## Supplementary Information

**Additional file 1.**

**Additional file 2.**

## Data Availability

The data underlying this article will be shared on reasonable request to the corresponding author.

## References

[1] WHO Coronavirus Disease (COVID-19) Dashboard. World Health Organization. Available from: https://covid19.who.int.

[2] Prado N, Rossi T, Chaves S, Barros SG, Magno L, Santos H (2020). The international response of primary health care to COVID-19: document analysis in selected countries. Cad Saude Publica.

[3] French National Health Insurance. Improve the quality of the health system and control over spendings. Propositions on costs and products of Health Insurance for 2021. 2020. Available from: https://www.ameli.fr/fileadmin/user_upload/documents/2020-07_rapport-propositions-pour-2021_assurance-maladie.pdf

[4] GIS EPI-PHARE. Use of medications out of the hospital in France during the COVID-19 pandemic: update after 8 weeks of lockdown and a week after lockdown. Pharmaco-epidemiological study on refunds data of the National System of Health Data. 2020. Available from: https://www.epi-phare.fr/app/uploads/2020/06/epi-phare_rapport_covid_3-1.pdf

[5] National Institute of Cancer. COVID-19 and cancer: a mobilisation and a continuous adaptation of the oncologic sector to avoid losses of chance. Available from: https://www.e-cancer.fr/Presse/Dossiers-et-communiques-de-presse/COVID-19-et-cancer-une-mobilisation-et-une-adaptation-continue-de-la-filiere-oncologie-pour-eviter-les-pertes-de-chance.

[6] Revil H., Blanchoz J-M., Olm C, Bailly S. Renounce to care during the lockdown. First results of the survey. Observatory of non-access to wrights and services (Odenore) 2020. Available from: https://odenore.msh-alpes.fr/documents/premiers_resultats_de_lenquete_du_brs_covid_-_decembre_2020_-_vd_1.pdf.

[7] Codagnone C, Bogliacino F, Gómez C. Longitudinal study on the effects of COVID 19 and lockdown in Italy, Spain, and United Kingdom. Open Evidence. 2020; [cited 12 June 2021]. Available from: https://open-evidence.com/wp-content/uploads/2021/05/20-05-28-COVID19-Open-Evidence-3-waves-EN-final.pdf.

[8] French Ministry of Health. Desktop for management not related to COVID-19. Published online on 8 April 2020. Available from: https://solidarites-sante.gouv.fr/IMG/pdf/soins-hors-covid-19.pdf.

[9] Dourgnon P, Naiditch M (2010). The preferred doctor scheme: a political reading of a French experiment of gatekeeping. Health Policy.

[10] Chaput H, Monziols M, Fressard L, Verger P, Ventelou B, Zaytseva A. More than 80% of general practitioners under 50 practice in a group. DREES. 2019; Available from: https://drees.solidarites-sante.gouv.fr/publications/etudes-et-resultats/plus-de-80-des-medecins-generalistes-liberaux-de-moins-de-50-ans.

[11] Chevillard G, Mousquès J, Lucas-Gabrielli V, Rican S (2019). Has the diffusion of primary care teams in France improved attraction and retention of general practitioners in rural areas?. Health Policy.

[12] Buchman S, Woollard R, Meili R, Goel R (2016). Practising social accountability: From theory to action. Can Fam Physician.

[13] Law n°2016–41 of January 26^th^ 2016 of modernization of the French health system (Title VI of the Public Health Code). 2016. Available from: https://www.legifrance.gouv.fr/jorf/article_jo/JORFARTI000031913246

[14] Saint-Lary O, Gautier S, Le Breton J, Gilbert S, Frappé P, Schuers M (2020). How GPs adapted their practices and organisations at the beginning of COVID-19 outbreak: a French national observational survey. BMJ Open.

[15] Thomas DR (2006). A general inductive approach for analysing qualitative evaluation data. Am J Eval.

[16] French National Health Insurance. Statistical data on the demographics of French health professionals. Available from: https://www.ameli.fr/l-assurance-maladie/statistiques-etpublications/donnees-statistiques/professionnels-de-sante-liberaux/ demographie/effectifs-et-densite.php

[17] Verhoeven V, Tsakitzidis G, Philips H, Van Royen P (2020). Impact of the COVID-19 pandemic on the core functions of primary care :will the cure be worse than the disease? A qualitative interview study in Flemish GPs. BMJ Open.

[18] Holmes EA, O'Connor RC, Perry VH, Tracey I, Wessely S, Arseneault L (2020). Multidisciplinary research priorities for the COVID-19 pandemic: a call for action for mental health science. Lancet Psychiatry.

[19] Grabovschi C, Loignon C, Fortin M (2013). Mapping the concept of vulnerability related to health care disparities: a scoping review. BMC Health Serv Res.

[20] National College of General Practice. Why and how register the social situation of an adult patient in general practice? 2014. Available from: https://lecmg.fr/wp-content/uploads/2019/02/doc_iss_02_04-1.pdf

[21] Oubaya N, Mahmoudi R, Jolly D, Zulfiqar AA, Quignard E, Cunin C (2014). Screening for frailty in elderly subjects living at home: validation of the Modified Short Emergency Geriatric Assessment (SEGAm) instrument. J Nutr Health Aging.

[22] Garnotel H, Ferry M, Chastang J, Ibanez G. GPs’ views on the collection of patients’ social situation in medical records. Exercer. (172):48–54.

[23] Tarrant C, Angell E, Baker R, Boulton M, Freeman G, Wilkie P, et al. Collecting and using diversity data in primary care. Responsiveness of primary care services: development of a patient-report measure – qualitative study and initial quantitative pilot testing. NIHR Journals. Library. 2014; Available from: https://www.ncbi.nlm.nih.gov/books/NBK263690/. 25642526

[24] Monziols M, Chaput H, Verger P, Scronias D, Ventelou B (2020). How did general practitioners operate during the Covid-19-related lockdown? DREES, Études et Résultats, 1150.

[25] Letrilliart L, Rigault-Fossier P, Fossier B, Kellou N, Paumier F, Bois C (2016). Comparison of French training and non-training general practices: a cross-sectional study. BMC Med Educ.

[26] Mousquès J, Daniel F. Group practice in multidisciplinary groups and centres generates productivity and expenses gains. Questions d’Economie de la Santé n°211. Inst Res Doc Health Econ Paris. 2015; Available from: http://www.irdes.fr/recherche/questions-d-economie-de-la-sante/211-l-impact-de-l-exercice-regroupe-pluriprofessionnel-sur-la-qualite-des-pratiques-des-medecins-generalistes.pdf.

[27] Offline HR (2020). COVID-19 is not a pandemic. Lancet.

[28] Desborough J, Dykgraaf SH, Phillips C, Wright M, Maddox R, Davis S, Kidd M (2021). Lessons for the global primary care response to COVID-19: a rapid review of evidence from past epidemics. Fam Pract.

[29] Bachelet M, Anguis M. Physicians by 2040: a younger, more female population and more often salaried. DREES, Études et Résultats. 2017;1011 Available from: https://drees.solidarites-sante.gouv.fr/sites/default/files/2020-08/er1011.pdf.

