# Supplementary material for: The challenge for general practitioners to keep in touch with vulnerable patients during the COVID-19 lockdown: an observational study in France
Source: BMC Prim Care. 2022 Apr 18;23:82. doi: 10.1186/s12875-022-01694-y (PMC9014789; doi:10.1186/s12875-022-01694-y)
Supplement: Supplementary file 1 — Additional file 1. [file 12875_2022_1694_MOESM1_ESM.docx]

**Appendix 1. List of the study partners.**

Members of the ACCORD network (Assembler, Coordonner, COmprendre, Rechercher, Débattreensoinsprimaires): the National College of Academic GPs (CNGE), the association SPP-IR (Multidisciplinarity in Primary Care, Innovation and Research), the French Society of General Practice (SFMG), the National Federation of Multidisciplinary Health Houses (AVECSanté), the Research Institute of Health Care Centres (IJFR), the unity RETINES (Risk, Epidemiology, Territory, INformations, Education in health) of University of Côte d’Azur, the National Association of Health Teams in Primary Care (ASALEE), the mission RESPIRE (EHESP) and the Midwifery Department of Versailles Saint Quentin University.

The partners of the ACCORD network who sent the link of the survey were the French Society of Generalist Care (SFTG), the National College of General Practice (CMG), the French Society of Documentation and Research in General Practice (SFDRMG), the French Society of General Practice (SFMG) and the French Association of Young Researchers in General Practice (FAYR-GP).
