# Supplementary material for: The challenge for general practitioners to keep in touch with vulnerable patients during the COVID-19 lockdown: an observational study in France
Source: BMC Prim Care. 2022 Apr 18;23:82. doi: 10.1186/s12875-022-01694-y (PMC9014789; doi:10.1186/s12875-022-01694-y)
Supplement: Supplementary file 2 — Additional file 2. [file 12875_2022_1694_MOESM2_ESM.docx]

Appendix 2. Practice location of the survey participants (n=3,012), in comparison to the general practitioners’ population in metropolitan France in 2019.

| n (%) | Study sample  n = 3,012 | GPs’ population in metropolitan France  n = 53,339 |
| --- | --- | --- |
| French regions  Auvergne-Rhône-Alpes  Bourgogne-Franche-Comté  Bretagne  Centre-Val de Loire  Corse  Grand Est  Hauts-de-France  Île-de-France  Normandie  Nouvelle-Aquitaine  Occitanie  Pays de la Loire  Provence-Alpes-Côte d’Azur  *Missing data* | 439 (16.3)  151 (5.6)  117 (4.4)  96 (3.6)  0 (0.0)  221 (8.2)  226 (8.4)  424 (15.8)  136 (5.1)  295 (11.0)  235 (8.7)  219 (8.1)  130 (4.8)  *323* | 6,774 (12.7)  2,240 (4.2)  2,880 (5.4)  1,760 (3.3)  267 (0.5)  4,694 (8.8)  4,907 (9.2)  8,161 (15.3)  2,507 (4.7)  5,547 (10.4)  5,494 (10.3)  2,934 (5.5)  5,174 (9.7)  *None* |

GP: general practitioner

* Data from the French health insurance system (CNAMTS) (2019)
